# Overweight but unseen: a review of the underestimation of weight status and a visual normalization theory

**DOI:** 10.1111/obr.12570

**Published:** 2017-07-21

**Authors:** E. Robinson

**Affiliations:** ^1^ Department of Psychological Sciences University of Liverpool Liverpool UK

**Keywords:** Body‐weight norms, obesity, visual adaptation, weight misperceptions

## Abstract

Although overweight and obesity are widespread across most of the developed world, a considerable body of research has now accumulated, which suggests that adiposity often goes undetected. A substantial proportion of individuals with overweight or obesity do not identify they are overweight, and large numbers of parents of children with overweight or obesity fail to identify their child as being overweight. Lay people and medical practitioners are also now poor at identifying overweight and obesity in others. A visual normalization theory of the under‐detection of overweight and obesity is proposed. This theory is based on the notion that weight status is judged relative to visual body size norms. Because larger body sizes are now common, this has caused a recalibration to the range of body sizes that are perceived as being ‘normal’ and increased the visual threshold for what constitutes ‘overweight’. Evidence is reviewed that indicates this process has played a significant role in the under‐detection of overweight and obesity. The public health relevance of the under‐detection of overweight and obesity is also discussed.

## Introduction

The prevalence of overweight and obesity is high in most countries in the developed world 1, 2. Of particular note has been the dramatic increase in obesity prevalence observed in recent times 3, 4. Because of this, obesity has now been recognized as a global crisis 5, 6 and is a well‐discussed matter of considerable public interest 7, 8. Here, it is argued that although obesity is now a widely discussed societal issue, a sizeable and diverse body of evidence has accumulated, which suggests that overweight and obesity often go ‘undetected’ and are under‐recognized conditions. This review first summarizes evidence for the under‐detection of overweight and obesity by drawing on research on self and parental perceptions of weight status and studies examining the ability of lay people and medical practitioners to visually identify overweight and obesity in others. Next, a ‘visual normalization’ theory to explain the under‐detection of overweight and obesity is outlined. This theory is based on evidence, which suggests that (i) evaluations about weight status are made relative to visual body‐weight norms and (ii) visual body‐weight norms are shaped by the size of bodies a person is frequently exposed to in his or her environment. Widespread increases in population adiposity have resulted in much frequent exposure to heavier body weights, and it is argued that this has altered visual perceptions of what constitutes a ‘normal’ weight, as well as shifting the visual threshold at which a person is identified as being overweight. This visual normalization process is argued to have played a causal role in the widespread under‐detection of overweight and obesity. Finally, the public health implications of the widespread under‐detection of overweight and obesity are considered. 1The content of this integrative review was informed by 2016/2017 literature searches. Where appropriate, I have also drawn on systematic reviews and meta‐analyses to inform the content of the review.


## Underestimation of self‐perceived weight status

Personal perceptions of weight status refer to how a person regards his or her own body weight and size, namely, whether he or she identifies himself or herself to be underweight, a healthy weight, overweight or obese. There have now been a large number of studies that have examined the correspondence between a person's actual weight status (typically defined using body mass index, BMI) and his or her self‐perceived weight status, assessed verbally or more commonly through the use of written or pictorial scales. A very consistent finding irrespective of how perception of weight status is measured is that although underestimation of weight status tends to be rare among individuals of ‘normal’ healthy weight (BMI of 18.5–24.9), a sizeable number of individuals with overweight (BMI of 25–29.9) fail to identify their weight status as being ‘overweight’ and instead identify their weight as being in the healthy weight range or ‘about right’ 9, 10, 11, 12, 13, 14. For example, in a recent study using 2013 data from a large nationally representative survey of UK respondents, 55% of adult men and 31% of women with overweight failed to identify their overweight weight status 12. Similarly, examining data from a nationally representative US study of over 16,000 participants, Yaesmiri *et al*. 10 report considerable underestimation of weight status (48% of men and 23% of women) among participants whose objective weight status was overweight. In comparison with individuals with overweight a smaller but still noteworthy percentage of individuals with obesity (BMI of ≥30) also believe their weight status to be ‘about right’ 10, 12, 15. In addition, there is evidence that in most cases, even if individuals with obesity do identify themselves as being ‘overweight’, a large number will fail to recognize the severity of their weight 16, 17. In line with this, a recent population‐based survey showed that the majority of UK adults with obesity do not identify that they are ‘very overweight’ and less than 10% correctly identify that they are ‘obese’ 18.

Although underestimation of self‐perceived weight status among individuals with overweight and obesity is frequently observed, it should be noted that some demographic factors play a role in explaining when underestimation is particularly likely to occur. Within the ‘overweight’ weight range (BMI of 25–29.9), the likelihood that a person underestimates his or her weight status is largest when his or her weight is at the lower end of this weight range, compared with the upper end of the overweight BMI range 14. However, even among those with a BMI that places them at the ‘upper’ of the overweight BMI range, a significant proportion of individuals still fail to identify that they are overweight 14. In addition, although some social and demographic patterning of personal weight status underestimation is observed (which is discussed later in this review), the tendency for individuals with overweight and obesity to underestimate their weight status is common across the lifespan and among different demographics. As well as being prevalent among adults, underestimation of self‐perceived weight status is common among children and adolescents 19, 20, 21, with a recent study of more than 70,000 US high school students showing that around one third of the respondents with overweight or obesity failed to correctly identify their weight status as being overweight 22. Underestimation of weight status among individuals with overweight and obesity is frequently observed in both the rich and the poor 23, 24, 25, as well as across a diverse range of ethnic groups 9, 26, 27.

The consistency by which studies show that substantial numbers of individuals with overweight fail to identify their weight status has recently been recognized in a systematic literature review 28. Moreover, recent work has also suggested that measurement error associated with the use of self‐reported data to determine objective weight status is likely to have resulted in the prevalence of weight status misperceptions among individuals with overweight and obesity being substantially underestimated in some previous studies 12. Thus, a wealth of research indicates that individuals with overweight and obesity often fail to accurately identify their weight status, and the prevalence of such misperceptions of weight status may be even larger than previously estimated.

## Parental underestimation of child overweight and obesity

A number of studies have shown that although parents of ‘normal’ healthy weight children rarely underestimate their child's weight status, parents of children with overweight or obesity often fail to recognize their child's weight status as being overweight 29, 30, 31, 32. The consistency of this finding is highlighted by a recent systematic review and meta‐analysis of studies examining the correspondence between parental perceptions of child overweight and anthropometric measurements 33. Lundahl and colleagues' analysis of over 60,000 children and their families showed that approximately one half of parents failed to identify their child's overweight or obese weight status 33. As is the case with self‐perceptions of weight status, there are factors that predict when parental underestimation of child overweight is particularly likely to occur. Firstly, the size of a child matters. Failure to identify child adiposity is more common if a child is overweight rather than obese 33, 34, 35, but still, a substantial proportion of parents of obese children incorrectly perceive their obese child's weight as being ‘about right’ 32, 35, 36, 37. Secondly, although parental underestimation of child weight status appears to be particularly pronounced for younger children (e.g. 2–5 years old) 33, 38, 39, high rates of parental underestimation (e.g. more than one third of parents underestimating) are still observed across older childhood and adolescence 30, 32, 38. Likewise, although some studies have indicated that fathers are particularly likely to underestimate the weight status of their overweight child, studies repeatedly show that mothers of overweight children also frequently fail to identify that their child is overweight or obese 40, 41. Thus, as is the case with personal misperceptions of weight status, although some demographic factors have been reported to be associated with a particularly high prevalence of parental underestimation of child overweight, the failure of parents to identify adiposity is widespread 33, 38.

## Failing to see overweight and obesity in others

Widespread personal and parental underestimation of overweight and obesity is consistent with the notion that overweight and obesity are under‐detected and often go unrecognized. A distinct literature suggests that under‐detection of overweight and obesity is also very common when both lay people and trained healthcare professionals judge the weight status of other people. This observation is in fitting with the notion that as a society, we may no longer know what constitutes an ‘overweight’ body size. Supporting evidence for this observation comes from a controlled study in which experienced and trainee doctors were asked to judge the weight status of photographed men 42. Although both experienced and trainee doctors were generally accurate at identifying the weight status of men who were of ‘normal’ healthy weight, there was a systematic underestimation of the weight status of men with overweight and obesity, resulting in a failure by experienced and trainee doctors to identify overweight and obesity more frequently than not 42. In actual and simulated consultation settings, a similar pattern of results is observed, whereby both adults and children with overweight and obesity are not recognized by medical professionals as being overweight or severely obese as they actually are 43, 44, 45, 46. For example, a large study of experienced physicians in Germany found that physicians accurately identified the weight status of less than 30% of patients with overweight and only around half of patients with obesity 43. Such is the consistency of this finding that the tendency for healthcare professionals to fail to identify overweight and obesity has been suggested to be a key factor explaining why weight loss treatment is provided only sparsely in healthcare settings: healthcare professionals do not think to weigh a patient or discuss weight management because their patients simply do not appear to be overweight or obese 43, 44, 47, 48.

The same pattern of under‐detection of overweight and obesity is also observed when lay people are asked to estimate the weight status of other people. Controlled studies have shown that male overweight and obesity are more likely to be underestimated than accurately perceived when weight status is judged using visual information alone (e.g. standardized photographic stimuli) and this effect has been observed in participants from Europe and the USA 49, 50. Likewise, a recent study showed that the weight status of women with overweight or obesity is systematically underestimated by lay people when judged using visual information alone 51. In further support, survey studies suggest that obese body weights are rarely visually identified as being obese until the objective body size being judged belongs to the upper end of class II obesity and above (extreme obesity) 52, 53. Although there has been no formal systematic literature of how frequently overweight and obesity go visually undetected by others, the studies reviewed here indicate that overweight and obesity are often visually under‐detected and the visual ‘threshold’ of what constitutes an ‘overweight’ body size is underestimated by both lay people and healthcare professionals.

## Surrounded by obesity and failing to see it

As has been suggested by others 14, 19, 43, 49, central to the proposed visual normalization theory is the notion that exposure to obesity results in the normalization of larger body sizes and it is this process that is a key contributor to the under‐detection of overweight and obesity. One prediction of this theory is that under‐detection should have become more common as the obesity epidemic has unfolded. In support of this, a number of longitudinal studies have documented that the tendency for individuals with overweight to fail to identify their adiposity and parental underestimation of child overweight have increased concurrently alongside population obesity prevalence 13, 14, 54, 55, 56. Johnson and colleagues examined self‐perceived overweight between 1999 and 2007 in the UK adults and found that during this period of population weight gain, the percentage of individuals with overweight or obesity underestimating their weight status increased. Burke and colleagues 54 reported conceptually similar findings in the US participants: from 1988 to 2004, a period that was associated with rapid increases in population‐level weight gain in the USA, men and women with overweight or obesity became less likely to identify their weight status as being ‘overweight’. A similar pattern of findings has been reported in longitudinal studies examining weight status misperceptions using other US data, among European adults and in studies that have examined temporal changes in parental underestimation of child overweight status over time 13, 55, 56. These findings indicate that as larger body weights have become more common, the tendency for overweight and obesity to go undetected has increased.

A further prediction of a normalization explanation of the under‐detection of overweight and obesity is that failure to identify overweight should be most common among people that are most frequently exposed to obesity on a daily basis. In support of this, a number of cross‐sectional studies suggest that living in a high obesity prevalence area is associated with an increased likelihood that a person underestimates his or her weight status and parental underestimation of child weight. Binkin and colleagues showed that residing in a high obesity prevalence region is associated with a higher likelihood of the US mothers failing to correctly identify their child as being overweight 57. Among school children, the likelihood that a child fails to identify his or her own overweight or obese status is greater if a large number of his or her classmates are obese 58. In a similar vein, overweight adolescents who report having larger friends are more likely to underestimate their weight status than those who report belonging to slimmer friendship networks 59. Likewise, being exposed to parents and/or peers of heavier body weight has been shown to be associated with a greater likelihood that children or adolescents fail to accurately identify that they themselves are overweight 19, 60. Environments in which obesity is ‘normal’ appear to promote the under‐detection of overweight and obesity in the self and others.

## Body‐weight norms and perceived weight status

The recalibration of visual body‐weight norms is a likely reason why increased obesity prevalence has caused the under‐detection of overweight and obesity. When individuals make evaluations about physical characteristics such as body size, they are often influenced by social comparison or ‘norm‐based’ information. A number of well‐supported theories highlight the importance of social comparison processes when making evaluations 61, 62, 63. Looking at the appearance of those around us provides us with a ‘standard’ or internal representation of what is normal. We then make evaluations in comparison with these types of internal standards or ‘norms’ 64, 65, 66. Thus, evaluations about whether a body size is ‘overweight’ are likely to be driven by a ‘norm’ comparison process, whereby the magnitude of a stimulus (the body size being evaluated) is compared with what a person believes to be a normal stimulus size (the evaluator's internal perception of what constitutes a ‘normal’ body size) 67, 68. In the current environment in which overweight and obesity are common, a norm‐comparison process therefore results in overweight and obese body weights appearing normal.

Consistent with a norm‐comparison explanation of weight status evaluations is the finding that personal perceptions of weight status appear to be dependent on a person's relative position in a population distribution. Regardless of whether they are objectively overweight, if a person's body size is statistically close to the ‘average’ weight of a population, it is unlikely they will recognize that their body size is overweight 69. Variants of this ‘normalization’ explanation have been offered to explain why increases in obesity prevalence have been associated with increased misperception of weight status 14, 19, 54, 69, but until recently, these suggestions have been based on observational data and not formally tested. A series of recent experimental studies lend direct support to a ‘norm‐comparison’ explanation of perceived weight status. In these studies, whether or not men and women with overweight or obesity were accurately identified by participants as being overweight was neatly predicted by the degree to which men's or women's body size deviated from the range of body sizes categorized perceptually as being ‘normal’ by most participants 51; if a woman with overweight had a body size that was perceptually categorized as being normal, her weight status was systematically underestimated. A person's evaluation of his or her own weight status has also been shown to be predicted by the extent to which he or she perceives his or her body size to deviate from that of an ‘average’ person's; believing that one's body size is similar or smaller than that of an average person's body size increases the likelihood that a person fails to identify that he or she is overweight 67. Likewise, experimentally manipulating the extent to which a person believes his or her body size is heavier or slimmer than ‘average’ has a causal effect on his or her self‐perceived weight status 67.

One 70 of the few qualitative studies 70, 71, 72 that has examined how parents of children with overweight or obesity judge their child's weight status provides further support to a normalization theory of weight status underestimation. Jones and colleagues 70 report that parents describe basing their opinions about their child's weight on visual comparison with how their child's weight compares with those of other children. This suggests that providing a child's weight does not appear to deviate from normality; parental detection of overweight will be unlikely. In support of this, a number of studies have shown that accurate parental identification of child overweight does not reliably occur until a child's weight is at the upper end of a population distribution 33, 73. This occurs presumably because it is not until a child's body size deviates substantially from perceived normality that it is evaluated as being overweight.

## Recalibration of visual body‐weight norms

Perception is shaped by previous experience, and because of this, increased obesity prevalence is likely to have recalibrated visual body‐weight norms. In other words, the size of a stimulus (e.g. body size) a person is used to seeing in his or her environment is likely to directly inform his or her visual perception of stimulus normality, a process that is frequently referred to as ‘visual adaptation’ 74, 75 or the ‘visual diet’ 68, 76. Because visual body‐weight norms will be similarly shaped by experience 77, 78, it is proposed that increased exposure to obesity will recalibrate the range of body sizes that are perceived by most people as being ‘normal’, as well as increasing the size at which a body is categorized as overweight. In support of this, when an overweight body is presented alongside other overweight bodies, as opposed to slender bodies, it appears thinner and is less likely to be judged as overweight 79, 80, presumably because it appears more normal. Recent experimental work provides the most direct experimental support to a normalization theory of weight status underestimation. A series of studies have shown that visual exposure to male obesity recalibrates perceptions of what constitutes a ‘normal’ male body size and this process causes men with overweight to be incorrectly perceived as being of ‘healthy’ weight 81, 82. Likewise, studies examining visual exposure to different female body sizes show that acute repeated exposure to larger body sizes affects perceptions of what constitutes a ‘normal’ or desirable body size for a woman 83, 84, 85 and results in the weight status of overweight women being underestimated 51. Outside of the laboratory, there is also evidence that an individual's visual perception of what constitutes a ‘normal’ body size is significantly larger if that individual interacts more frequently with social contacts who are obese 86.

There are also a number of observational studies that provide complementary but indirect support to the premise that exposure to obesity alters visual perceptions of body weight. In a large cross‐cultural study of over 800 adults from the USA, Mexico, Korea, Ukraine and Tanzania, Johnson and colleagues found that individual differences in the visual perceptual threshold for overweight are strongly associated with local population prevalence of obesity 87; a higher local prevalence of obesity was associated with a larger body size visual threshold for what constituted overweight. A recent cross‐cultural study reports results in fitting with Johnson and colleagues' observations; participants from a country with high obesity prevalence (USA) were more likely to visually underestimate the weight status of obese men than participants from countries with lower obesity prevalence 50. Moreover, within a population (UK), it has been shown that young adults who tend to socialize with overweight peers are particularly poor at visually identifying overweight and obese body weights in others 49. Data from a migration study also lend support to the hypothesis that exposure to obesity can recalibrate visual perceptions of personal body size. Japanese women migrating to an environment in which obesity was more prevalent (USA) showed a change in perceived body size: after living in their new obesity‐prevalent environment for 2 months, Japanese women perceived their body size as being smaller 88. Thus, there is accumulating evidence that points to visual exposure to larger body sizes having recalibrated perceptions of what ‘normal’ body sizes look like and this process being responsible for the under‐detection of overweight and obesity.

## Social patterning of personal underestimation of overweight and obesity

Although the under‐detection of overweight and obesity is common, some individual‐level characteristics are associated with a person being more or less likely to accurately identify that he or she is ‘overweight’. For example, individuals with overweight who are aware of the medical guidelines for what constitutes overweight and obese body weights are less likely to underestimate their weight status than those who are less knowledgeable 17, 89. Three other factors very consistently associated with personal misperception of overweight and obesity are gender, ethnicity and socioeconomic status. Accurate perception of personal overweight and obesity weight status is more likely to occur in women than in men 10, 13, 14, 17, in Caucasians than in people of African descent 24, 26, 90 and among individuals with higher socioeconomic status 24, 90, 91. There are likely to be a number of contributing factors to this social patterning, some of which will not be directly relevant to visual normalization (e.g. education or health literacy). However, visual normalization may also be important in explaining some of the social patterning of the under‐detection of overweight and obesity. In developed countries, the prevalence of obesity is higher in low socioeconomic groups and among people of African descent communities, as opposed to wealthier Caucasian communities 92, 93, 94, which results in more frequent visual exposure to larger body sizes. This level of increased exposure to obesity would be predicted to affect the range of body sizes that socioeconomically disadvantaged and people of African descent perceive as being ‘normal’, which in turn would explain why underestimation of overweight and obesity is more common in these demographics. Indeed, the body sizes that people of African descent and those of lower socioeconomic status report as being ‘normal’ or ‘ideal’ do tend to be significantly larger than body size norms reported by Caucasian individuals or those of a higher socioeconomic status 95, 96, 97. In a similar vein, personal underestimation of overweight and obesity has been reported to be particularly common among Hispanic individuals, an ethnic group with very high rates of obesity and a tendency to perceive larger body sizes as being more normal 26, 98, 99.

Although there are some gender differences in obesity prevalence in developed countries, these differences tend to be relatively small and less striking than those reported as a consequence of race or social class 1, 100, 101. Thus, differences in obesity prevalence appear to be less suited to explain gender differences in the underestimation of overweight and obesity. However, a substantial body of research does suggest that women, but not men, in developed countries are very frequently exposed to slender same‐sex bodies through popular media 102, 103, 104. Therefore, the tendency for women to be exposed to slender female bodies in the media may cause the perception of what constitutes a ‘normal’ female body size to be smaller than that of a ‘normal’ male body size 105 because of ‘internalization of thin ideals’. According to the proposed normalization theory, exposure to thin ideals would in part counteract visual exposure to obesity and make underestimation of weight status less common in women, as opposed to men. Indeed, this is well observed in large survey studies of self‐perceived weight status 10, 13, 17. Additionally, in a recent study from our laboratory, we found that visual underestimation of the weight status of others is less common when judging women, as opposed to men 70. The proposition that the size of bodies that women are exposed to in the media may explain why there are gender differences in weight misperceptions is also in fitting with experimental data showing that exposure to media that presents very slender female bodies causes women to perceive their body size to be heavier 106, 107. The extent to which the proposed visual normalization theory explains social patterning of weight misperceptions (e.g. gender, ethnic and socioeconomic differences) would now benefit from direct testing.

## Unanswered questions about the underestimation of weight status

I have proposed that visual normalization is likely to explain a substantial amount of the under‐detection of overweight and obesity. However, it is of importance to note that evaluations of weight status are likely to have both visual and attitudinal components. The majority of studies that have assessed personal and parental perceptions of weight status ask participants how they would describe their own/their child's weight. Thus, the method adopted in these studies does not tell us whether underestimation of weight status is caused by participants' visual perception of body size (‘My child's weight looks normal, not overweight’) or a more affective appraisal of a participant's attitude towards their weight (e.g. ‘I don't feel overweight’). However, a number of studies that have asked participants to use visual rating scales (e.g. ‘which is most similar to your/your child's body size’) corroborate that underestimation of overweight and obesity is very common 11, 19, 33, 53, a finding that is in fitting with a visual account of weight status underestimation. Further work designed to directly examine the relative contributions of visual perception versus attitudes in explaining personal and parental underestimation of weight status would now be informative, as it would test the validity of the proposed visual normalization theory of the under‐detection of overweight and obesity. In addition, although the tendency for weight status to be underestimated was the focus of the present review, there is also literature on the underestimation of absolute body weight (e.g. in kilograms). Recent work has suggested that visual biases may also be important in explaining why heavier absolute body weights tend to be underestimated 68. For example, ‘contraction bias’ relates to the natural tendency for visual stimuli that are larger than average to be prone to size underestimation 68, 108. Thus, ‘contraction bias’ may also in part explain underestimation of weight status among individuals with overweight and obesity. Understanding whether overlapping processes explain both the underestimation of absolute body weight and weight status would now be informative.

## Public health implications of the under‐detection of overweight and obesity

It has been widely assumed that large numbers of individuals failing to identify that they are overweight or obese is a public health concern on the grounds that a failure to identify overweight or obesity will be a hindrance to weight management or intervention efforts 10, 14, 21, 53, 54, 108, 109; if one fails to recognize that they are overweight, then they are presumed to be unlikely to take the correct steps to address this. This line of reasoning bleeds over into public health intervention efforts that are designed to improve detection and educate people that they are ‘overweight’ 110. In support of this line of reasoning, a number of studies clearly show that whether or not a person with overweight or obesity correctly identifies his or her weight status is associated with greater weight loss intentions; people who recognize they are overweight are more likely to report a greater desire to lose weight and intentions to diet 10, 69, 111. Although this line of reasoning for why the under‐detection of overweight and obesity could be detrimental to weight management makes intuitive sense 10, 14, more recent research has started to question this commonly held assumption. Furthermore, although an ‘ignorance is damaging’ view point has been endorsed by researchers for some time, supporting evidence that largely comprises self‐reported intention measures, which have substantial limitations. For example, self‐identification of being overweight is associated with greater reported weight loss intentions in cross‐sectional analyses, but this does not tell us whether these intentions translate to successful weight management.

There is considerable stigma attached to the labels of being ‘overweight’ or ‘obese’ 112, 113, 114. Thus, individuals who recognize that they are overweight will be aware that they are part of a widely stigmatized and derogated social group. This is of relevance, because identifying with a devalued social group is likely to be stressful and damaging to self‐worth 115, 116. Therefore, because of the stigma attached to being ‘overweight’, accurate personal identification of ‘overweight’ or ‘obesity’ may actually be to the detriment of an individual's psychological well‐being. Cross‐sectional studies in adolescent and adult populations lend some support to this, as they show that accurately identifying one's own weight status as being ‘overweight’, as opposed to failing to identify oneself as overweight, is associated with a range of poor mental health outcomes, including greater risk of depression 117, 118, 119 and reduced quality of life 120. A number of theoretical models also suggest that the psychological burden of identifying as ‘overweight’ could manifest itself in unhealthy behaviours that end up promoting weight gain and exacerbating obesity 121, 122, 123. In support of this, across three large nationally representative samples of the US and UK adults, individuals who identified that they were overweight went on to gain more weight than those who did not identify that they were overweight 124. This same basic finding has also been observed among adolescents and young adults; overweight adolescents and young adults who correctly identify that they are overweight are at a greater risk of future elevated weight gain compared with those who fail to identify that they are overweight 125, 126, 127. How these findings relate to the earlier discussed observation that a person identifying his or her weight status as being overweight is associated with a greater desire to lose weight is important to consider. One possibility is that although self‐identification of overweight promotes a greater desire to lose weight, the negative psychological consequences of identifying as part of a stigmatized social group compromise effortful self‐regulation and/or promote counterproductive weight control behaviours that eventually result in further weight gain 128. A comprehensive analysis of all evidence surrounding the public health implications of the under‐detection of overweight and obesity is beyond the scope of the current review. Yet it is clear that there is an emerging body of research that challenges the traditional view point that failing to identify one's own weight as being ‘overweight’ will be to the detriment of that person's health.

## Conclusions

Evidence that suggests that overweight and obesity go under‐detected was reviewed; large numbers of individuals with overweight or obesity fail to recognize that they are overweight or obese, and people regularly fail to identify others as being overweight or obese. A visual normalization theory of the underestimation of overweight and obesity was proposed; because larger body sizes are now common, this is likely to have caused a recalibration to the range of body weights that are perceived as being ‘normal’ and increased the visual threshold for what constitutes an ‘overweight’ body size. It is proposed that this visual recalibration process has played a significant role in the under‐detection of overweight and obesity. Although failures to identify overweight or obesity have been presumed by many to be damaging to health, here, evidence was reviewed, which indicates that this presumption may be incorrect.

## Conflict of interest statement

No conflict of interest was declared.
